# Investigation of Possible Correlation between *Giardia duodenalis* Genotypes and Clinical Symptoms in Southwest of Iran

**Published:** 2013

**Authors:** Abdollah RAFIEI, Elham Sadat ROOINTAN, Ali Reza SAMARBAFZADEH, Ali Akbar SHAYESTEH, Ahmad SHAMSIZADEH, Mahdi POURMAHDI BORUJENI

**Affiliations:** 1 Parasitology Department, Faculty of Medicine, Ahvaz Jundishapur University of Medical Sciences, Ahvaz, Iran; 2 Infectious and Tropical Diseases Research Center, Ahvaz Jundishapur University of Medical Sciences, Ahvaz, Iran; 3 Virology Department, Faculty of Medicine, Ahvaz Jundishapur University of Medical Sciences, Ahvaz, Iran; 4 Department of Internal Medicine, Imam Khomeini Hospital, Faculty of Medicine, Ahvaz Jundishapur University of Medical Sciences, Ahvaz, Iran; 5 Department of Pediatric, Abozar Hospital, Faculty of Medicine, Ahvaz Jundishapur University of Medical Sciences, Ahvaz. Iran; 6 Department of Food Hygiene, Faculty of Veterinary Medicine, Shahid Chamran University, Ahvaz, Iran

**Keywords:** *Giardia duodenalis*, Glutamate dehydrogenase (*gdh*), Semi-nested PCR, PCR-RFLP, Iran

## Abstract

**Background:**

*Giardia duodenalis* is one of the most important human enteric parasites throughout the world. Clinical symptoms of this parasite vary from asymptomatic infection to chronic diarrhea. Still it is not clear, whether different types of pathogenesis are due to different strains of organism or to variable host factors. The purpose of this study was to investigate possible correlation of clinical symptoms with assemblages among symptomatic and asymptomatic cases collected from southwest of Iran.

**Methods:**

Fecal samples were collected from 100 symptomatic and asymptomatic cases, which were positive for *G. duodenalis*. The samples were subjected to semi-nested PCR and RFLP for *gdh* gene.

**Results:**

Among symptomatic patients, 54% had mixed genotypes AII and BIII, 28% and 18% of samples indicated assemblages BIII and AII, respectively. In contrast, among asymptomatic cases, 64%, 26% and 10%samples had mixed genotypes, BIII and AII assemblages, respectively. Statistical analysis using Chi- Square test showed that there was no significant correlation between assemblage and clinical symptoms in current study.

**Conclusion:**

High prevalence of mixed infection in both groups may affect this conclusion, therefore further study in more details are necessary to clarify these finding. Additionally, it is important to carry out investigations regarding human host factors as well.

## Introduction

*Giardia duodenalis* is a protozoan parasite of the small intestine found in human and a wide range of mammalian hosts (1, 2). It is estimated that in Asia, Africa and Latin America, about 200 million peoples are now suffering from giardiasis with some 500,000 new cases reported annually. The prevalence of *Giardia* infection has been reported 5-23 percent in different urban and rural parts of Iran (3, 4). Clinical symptoms of *G. duodenalis* infection are highly variable and some patients develop clinical giardiasis while others remain asymptomatic (5, 6). Symptomatic cases usually are accompanied with weakness, weight loss, occasionally watery diarrhea, funky stool, steatorrhea, abdominal cramp, bloating, belching, nausea, vomiting and malabsorption syndrome (7). The risk factors for giardiasis are poorly understood and undoubtedly include host factors and may be strain variation of the parasite (6).

Molecular studies such as PCR-RFLP and sequence analysis of housekeeping genes have shown that *G. duodenalis* is a species complex comprising eight major assemblages (A to H) (6, 8–10). According to available data assemblages A and B have been reported in human (8, 11). Assemblage A has been classified into subgroups I and II (8). Assemblages B consist of two subgroups III and IV. Assemblages AII and BIV appears to be human-specific (11). Genotyping techniques such as PCR-RFLP are used to be a simple, sensitive and powerful analytical tool and it has been successfully used by a number of researchers for *Giardia* genotyping and *gdh* (glutamate dehydrogenase) gene is proving useful for genotyping (12, 13). Recently a few and insufficient studies have been performed to determine the genotype and its correlation with clinical symptoms but they have had conflicting results. Assemblage A was more prevalent in asymptomatic infection (14, 15), but it can cause clinical giardiasis (16–19). There was no correlation between assemblages and clinical symptoms (20–22). Current research designed to investigate possible correlation of *G. duodenalis* assemblages with clinical symptoms among human infected cases.

## Materials and Methods

### Samples collection and purification of cysts

This study was carried out from September 2011 to July 2012. Fecal samples were collected from one hundred human cases of *G. duodenalis*. The samples were collected from human cases less than 15 years old (as children) and more than 15 years old as adults, referred to Ahvaz health centers, southwest of Iran. These samples were divided into two groups: 50 fecal samples from symptomatic patients (25 fecal samples from adult patients, 25 from children suffering from giardiasis). In addition, 50 fecal samples were collected from asymptomatic cases divided into two groups as adults and children. The study population included 65 males and 35 females, ranging from 4 to 65 years old. A questionnaire was filled for all cases compromising demographic information and clinical symptoms including: fever, abdominal pain, abdominal cramps, flatulence, weight loss, nausea, vomiting and fatty diarrhea. Patients who had more than four of these signs or symptoms were considered as symptomatic and cases that do not have any signs and symptoms were considered as asymptomatic. *All of symptomatic children and adults cases were visited by* pediatric *or* gastroenterologist respectively, and all suspected cases with etiology other than giardiasis were excluded. The fecal samples were analyzed by wet smear stained with Lugol's iodine, formalin ether (23). All symptomatic patients who were *infected with other intestinal parasites or bacterial infection* were also excluded from the study. The cysts were purified and concentrated from the faeces by sucrose density gradient centrifugation and washed with sterile distilled water and then stored at -20 °C until used (24). The intensity of infection was estimated by average cyst count per high power field (HPF/ ×40) of light microscope. Samples scoring were divided into three categories: 1-5 (1+), 6-10 (2+) and more than 10 cysts (3+).

### DNA extraction

Prior to DNA extraction, cysts were freeze-thawed ten times at -80 °C and + 80 °C. DNA was extracted from purified samples by using the commercial QIAamp DNA Stool Mini Kit (Qiagen, Germany) according to the manufactures protocol. DNA samples were preserved at -20 °C until used.

### PCR amplification

The amplification of the *gdh* gene (432 bp) was performed as a semi-nested PCR with a external forward primer GDHeF (TCA ACG TYA AYC GYG GYT TCC GT), internal forward primer GDHiF (CAG TAC ACC TCY GCT CTC GG), and reverse primer GDHiR (GTT RTC CTT GCA CAT CTC C as described by Read et al. (13). The primers were tested with positive DNA control. The positive control *G. deudenalis* subgroup was generously donated by Kerman University of Medical Sciences. Distilled water used as negative control. The PCR reaction mixture comprised 5 µl genomic DNA, 5 µl of 10X buffer (Fermentas, Lithuania), 1.5 µl (50mM) MgCl2, 1 µl (10 mM) dNTP mix, 0.3 µl (5U/ µl) Taq DNA polymerase (Fermentas, Lithuania), and 1 µl (12.5) pmol of each primer. The reactions were performed in 50 µl valumes. GDHeF and GDHiR were used in the primary PCR reaction. One microliter of PCR product from the primary reaction was added to the secondary PCR containing primers GDHiF and GDHiR. The DNA was amplified using iCycler, BioRad Thermal Cycler under the following condition: 1 cycle of 94 °C for 3 min, 56 °C for 1 min and 72°C for 2 min, followed by 35 cycles, 94 °C for 1min, 56 °C for 20 s and 72°C for 45 s. A final extension of 72 °C for 7 min and a 20 °C hold was used. The PCR products were electrophoresed on ethidium bromide stained 1% agarose gels (Roche, Germany).

### PCR-RFLP of region of gdh gen

RFLP analysis was carried out by digesting 10 µl of PCR product. It was added to 1 X enzyme buffer, and 2 µl (10 U/ µl) *BspL1* (Fermentas, Lithuania) or 2 µl (10 U/ µl) *Rsa1* (Roche, Germany) for 16 h at 37 °C. The final valumes of *Rsa1* and *BspL1* were 25 and 30 µl, respectively. The *BspL1* digestion was used for the distinction between AI or AII and B assemblages. *Rsa1* digestion distinguished between subtypes BIII and BIV. Restriction fragments were separated in 3% high resolution grade agarose (Roche, Germany) stained with ethidium bromide. A 50 bp DNA ladder (Fermentas, Lithuania) was used as a size marker (13).

### Statistical analysis

All data processing was carried out by SPSS software version 16. Chi square test was used to evaluate the relationship between variables. Mann-Whitney U test was used to investigate the relationship between intensity of infection and clinical symptoms. Logistic regression was used to calculate the odds ratio and 95% confidence interval. α = 0.05 were considered for statistical analysis.

## Results

**Fig. 1:**
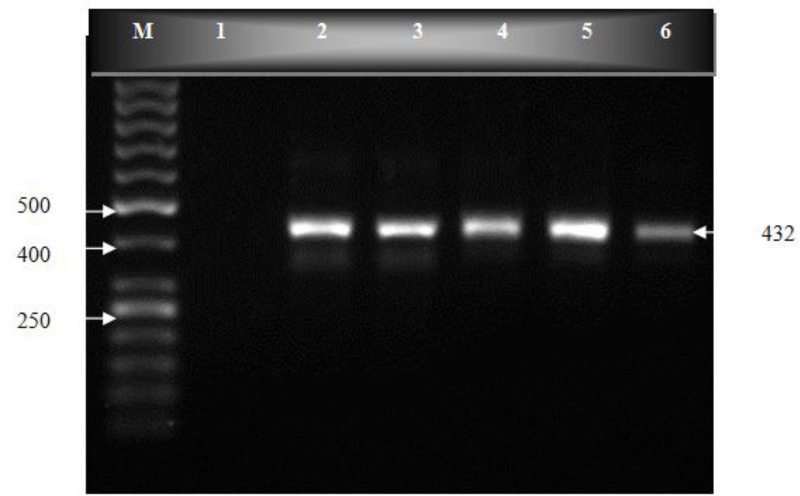
PCR products on an ethidium bromide-stained 1% agarose gel. lane M, molecular weight marker (50 bp); lane 1, negative control; lane 2, positive control; lanes 3-6, PCR products from clinical samples

**Fig. 2:**
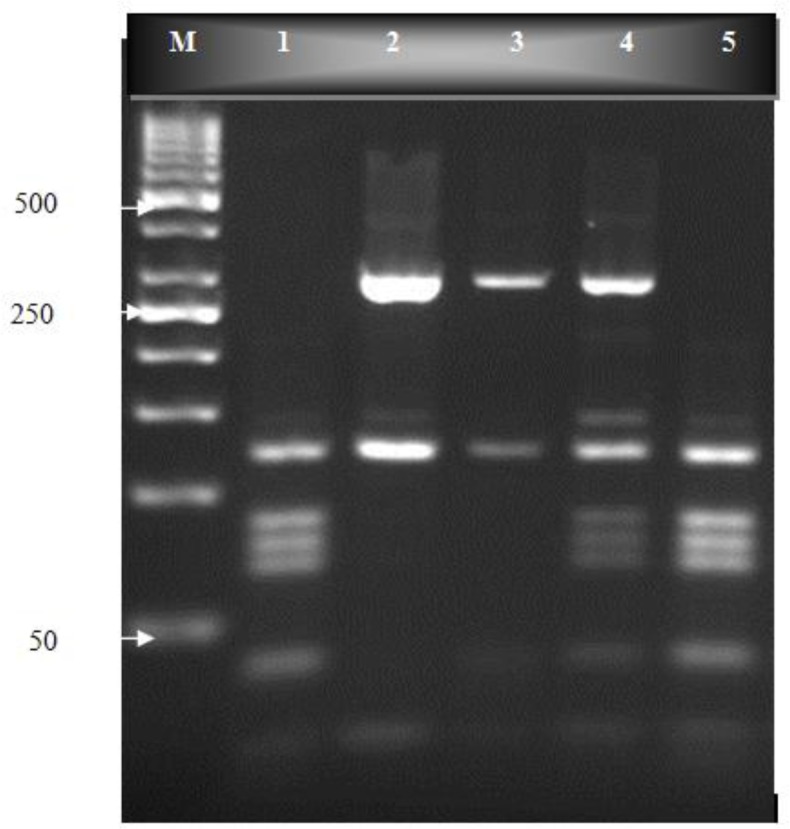
*Bspl1* digestion of *gdh*-PCR products on an ethidium bromide-stained 3% high resolution grade agarose gel. Lane M, molecular weight marker (50 bp); lane 1, *G. duodenalis* positive control (genotype AII), lanes 2-3, genotype B; lane 4, mixed genotype AII and B; lane 5, genotype AII

**Fig. 3:**
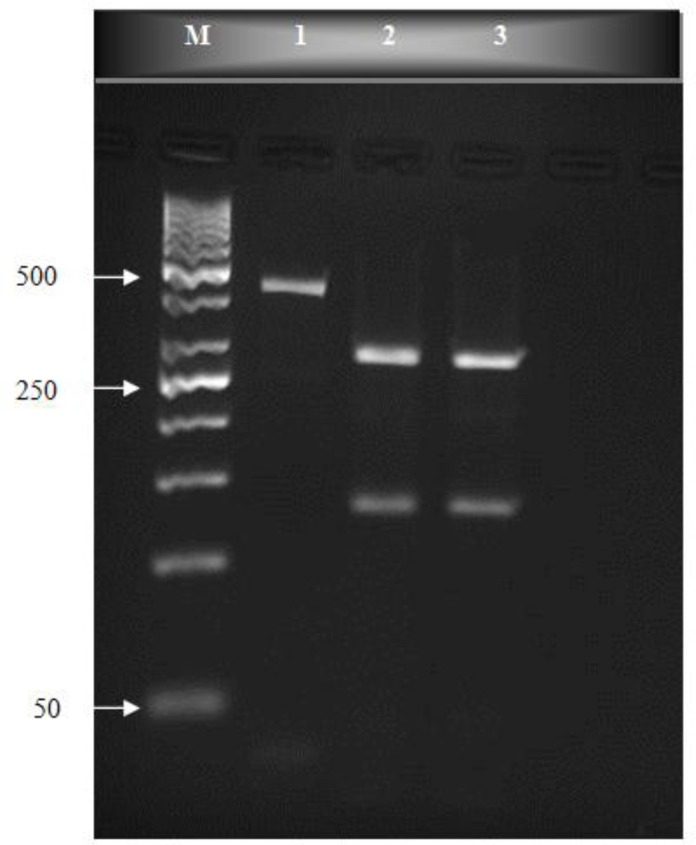
*Rsa1* digestion of *gdh*-PCR products on an ethidium bromide-stained 3% high resolution grade agarose gel. lane M, molecular weight marker (50 bp); lane 1, PCR products (432 bp fragment); lanes 2-3, genotype B group BIII

One hundred human fecal samples were amplified in semi-nested PCR (Fig. 1). PCR products were cut by *BspL1* and *Rsa1* restriction endonuclease, and the RFLP patterns of the digested products were studied (Figs. 2 and 3). The predicted fragment sizes are shown in Table 1. Fragments less than 50 bp were not included in the analysis, as they could not be reliably resolved on the gel and because the assemblages could be distinguished without the need to analyze these small bands.

**Table 1:** Predicted fragment sizes (bp) of *G. duodenalis* assemblages after digesting with *BspL1* and *Rsa1*

Assemblage	Enzyme	Predicted fragment sizes	Diagnostic genotyping profile
**AI**	*BspL1*	16, 18, 39, 87, 123, 149	90, 120, 150
**AII**	*BspL1*	16, 18, 39, 72, 77, 87, 123	40,70, 80, 90, 120
**B**	*BspL1*	18, 39, 123, 291	120, 290
**BIII**	*RsaI*	2,133, 297	130, 300
**BIV**	*RsaI*	12, 430	430

Fifty nine percent had mixed genotypes AII and BIII, defined by the presence of DNA bands at 40, 70, 80, 90, 120 and 290 bp. Twenty seven (27%) cases had assemblages BIII, defined by the presence of DNA bands at 130 and 300 bp and fourteen (14%) cases had assemblage AII, determined by the presence of DNA bands at 70, 80, 90 and 120 bp (Figs. 2, 3 and Table 1). Among symptomatic samples 27 (54%) cases contained mixed genotypes, 14 (28%) samples contained assemblage BIII and 9 (18%) samples indicated assemblage AII. On other hand among the 50 asymptomatic cases 32 (64%) samples had mixed genotypes AII and BIII, 13 (26%) samples were BIII and 5 (10%) samples showed assemblage AII.

There was no statistically significant correlation between assemblages and clinical symptoms (κ^2^=1.6, df=2, *P*=0.45). Intensity rate of infection in symptomatic patients (median: 3.55) was also more significantly than the asymptomatic patients (median: 2.4) (*P*=0.013).

## Discussion

Human giardiasis is a global disease that is caused by two major genetic assemblages A and B, of *G. duodenalis* (1). In current study, assemblage BIII was detected in 28% and 26% of symptomatic and asymptomatic patients and assemblage AII was detected in 18% and 10% of the same samples. Studies conducted in different parts of Iran indicated, that the most prominent types of assemblages were AII and BIII but with different prevalence rates (25–29). Clinical symptoms of this parasite are highly variable. Host factors and parasite strains are probably involved in different types of pathogenesis (5, 30). Studies on the correlation between assemblages and clinical symptoms have controversial results.

In our study, there was no correlation between the clinical symptoms and assemblages. These results correspond with those from studies carried out in Fars Province in southern of Iran by Sarkari et al. (28). They mentioned that assemblages A and B caused similar clinical symptoms. In contrast, in Kerman, central south of Iran, assemblage B was more significantly common among symptomatic patients (26). Assemblage B was equally distributed among symptomatic and asymptomatic patients and there was no correlation between assemblage B and clinical symptoms (21). Similarly, there was no relation between clinical presentation and genotypes of *G. duodenalis* in Brazilian children (20). There was no significant correlation between assemblages and clinical symptoms in older ages greater than 5 years in Spain (31).

In contrast, in Saudi Arabia, Hamdan reported that there was a correlation between assemblage B and symptomatic infection (32). Similarly, Mohammed Mahdy et al. (15) and Pelayo et al found that assemblage B was more significantly common among symptomatic patients (33). Assemblage B was more present in symptomatic patients (34). In other hand, some researchers reported correlation between assemblage A and symptomatic infection, and assemblage B with asymptomatic cases (16–19). In our study, symptomatic patients had more intensity rate of cysts than asymptomatic cases which may indicate higher parasite activity in symptomatic patients.

To our best knowledge, this is the first study that divided studied populations into children and adults in equal numbers compromising symptomatic and asymptomatic groups. Some earlier studies also reported a similar dual infection, but according to our literature review, it seems that the rate of mixed infection with genotypes AII and BIII, in our report is higher than others (25, 28, 31, 32, 35–39). This higher rate of dual infection among symptomatic and asymptomatic cases which is not in agreement with most previous investigation may play a role as a confounding variable for such study. We do not have a clear explanation for this higher rate of mixed infection in the region. It seems further study are needed to provide a better interpretation of the occurrence of mixed infections. Additionally, it is important to carry out studies regarding human host factors and possible correlation with clinical symptoms.

## Conclusion

According to current results and previous studies any correlation between giardiasis and assemblages remains unclear and further researches with more details are needed regarding clear interpretation.
